# Municipal solid waste incineration bottom ash in concrete : A systematic review and meta-analysis

**DOI:** 10.1038/s41598-025-23774-6

**Published:** 2025-11-14

**Authors:** Sivayogaraj A, Elavenil S

**Affiliations:** School of Civil Engineering, Vellore Institute of Technology-Chennai Campus, Chennai, India

**Keywords:** Municipal solid waste incineration bottom ash, Supplementary cementitious materials, Sustainability, Replacement, PRISMA, Meta-analysis, Engineering, Environmental sciences, Materials science

## Abstract

Municipal Solid Waste Incineration Bottom Ash (MSWIBA), a byproduct of waste-to-energy (WtE) facilities, has emerged as a promising candidate for use both as a sustainable aggregate replacement in concrete and as a supplementary cementitious material (SCM). This systematic review and meta-analysis, following Preferred Reporting Items for Systematic Reviews and Meta-Analyses (PRISMA) 2020 guidelines, evaluates its mechanical, durability, and environmental impacts. A literature search (2000–2025) across PubMed, Scopus, Web of Science, Google Scholar, and Engineering Village identified 38 studies with 312 experimental groups. Random-effects meta-analysis showed that MSWIBA replacement up to 20% results in a modest reduction in compressive strength (Standardized Mean Difference [SMD] = − 0.42, 95% Confidence Interval [CI] − 0.58 to − 0.26, *p* < 0.001), with substantial heterogeneity (I^2^ = 78%). Tensile strength (SMD = − 0.48, 95% CI − 0.68 to − 0.28, *p* < 0.001) and flexural strength (SMD = − 0.52, 95% CI − 0.74 to − 0.30, *p* < 0.001) also decrease moderately. Durability outcomes, including chloride penetration (SMD = 0.33, 95% CI 0.12 to 0.54) and freeze-thaw resistance (SMD = − 0.41, 95% CI − 0.72 to − 0.10), show mixed results due to limited data. MSWIBA reduces landfill use by saving 100 kg CO_2_ per ton of concrete and conserves natural aggregates, but variability in composition, potential heavy metal leaching, and reduced mechanical properties at higher replacements (> 20%) pose challenges.

**Supplementary Information:**

The online version contains supplementary material available at 10.1038/s41598-025-23774-6.

## Introduction

Global municipal solid waste (MSW) generation reached 2.24 billion tons in 2020, projected to increase to 3.88 billion tons by 2050^1^. The exponential growth in global population and urbanization has led to surging quantities of municipal solid waste (MSW), intensifying the need for efficient waste management and sustainable building practices^2^. Incineration is increasingly employed as a waste-to-energy (WtE) strategy, generating by-products such as Municipal Solid Waste Incinerated Bottom Ash (MSWIBA), Municipal Solid Waste Incinerated Fly Ash (MSWIFA) and Air pollution control residues (APCR)^3^. The construction industry faces pressure to adopt sustainable practices amid dwindling natural resources and landfill capacity^4^. MSWIBA represents approximately 80–90% of the total residue from municipal waste incineration processes, generating millions of tons annually worldwide^5^. Traditional disposal methods, including landfilling, are becoming increasingly unsustainable due to environmental concerns and limited landfill capacity^6^.

The incorporation of MSWIBA into concrete production offers a promising solution for waste valorization while potentially reducing the demand for cement and natural aggregates at optimum dosage levels^2,7^. MSWIBA contains aluminosilicate-rich phases with latent pozzolanic behavior that can be harnessed in cementitious applications^8^. MSWIBA potentially reducing CO_2_ emissions by up to 10–15% per ton of cement replaced^9,10^. However, the heterogeneous nature of MSWIBA, varying chemical composition, and potential presence of contaminants present challenges for its widespread adoption in concrete applications^11,12^. Previous studies have investigated various aspects of MSWIBA utilization in concrete, including mechanical properties, durability characteristics, and environmental implications^13,14^. They have also highlighted the chemical similarity of MSWIBA with conventional Supplementary Cementitious Materials (SCM) such as fly ash, silica fume and blast furnace slag^15^. Nevertheless, MSWIBA remains underutilized due to concerns related to heavy metal leaching and compositional variability^16,17^. Proper pre-treatment methods such as weathering, washing, alkali activation, and carbonation have been shown to mitigate environmental risks^18,19^.

However, results have been inconsistent, and no comprehensive systematic review has quantified the overall effectiveness of MSWIBA utilization in concrete through meta-analysis^20^. There is a growing need for systematic reviews and meta-analyses to critically assess materials used in cement replacement^20,21^. Identifying research gaps in this domain depends on evaluating how far current studies can be extended to address new or unresolved questions^22^. This process relies heavily on constructing a robust foundation through comprehensive literature reviews, which synthesize existing findings within relevant contexts^23^. However, many conventional narrative reviews are often limited by bias, insufficient empirical data, and a lack of methodological rigor^24^. As a result, they frequently fall short in providing reliable, evidence-based conclusions that support sound decision-making^25^.

This review evaluates MSWIBA as both a cement replacement (due to its pozzolanic properties) and an aggregate replacement (due to its physical characteristics), comparing its performance to established SCMs like fly ash and ground granulated blast-furnace slag (GGBS). This systematic review aims to synthesize available evidence on MSWIBA utilization in concrete, evaluate the quality of existing research, and provide quantitative estimates of the effects on concrete properties through meta-analysis following the updated guidelines and protocols stated by PRISMA 2020 statement^26^.

### Research methodology

**Fig. 1:**
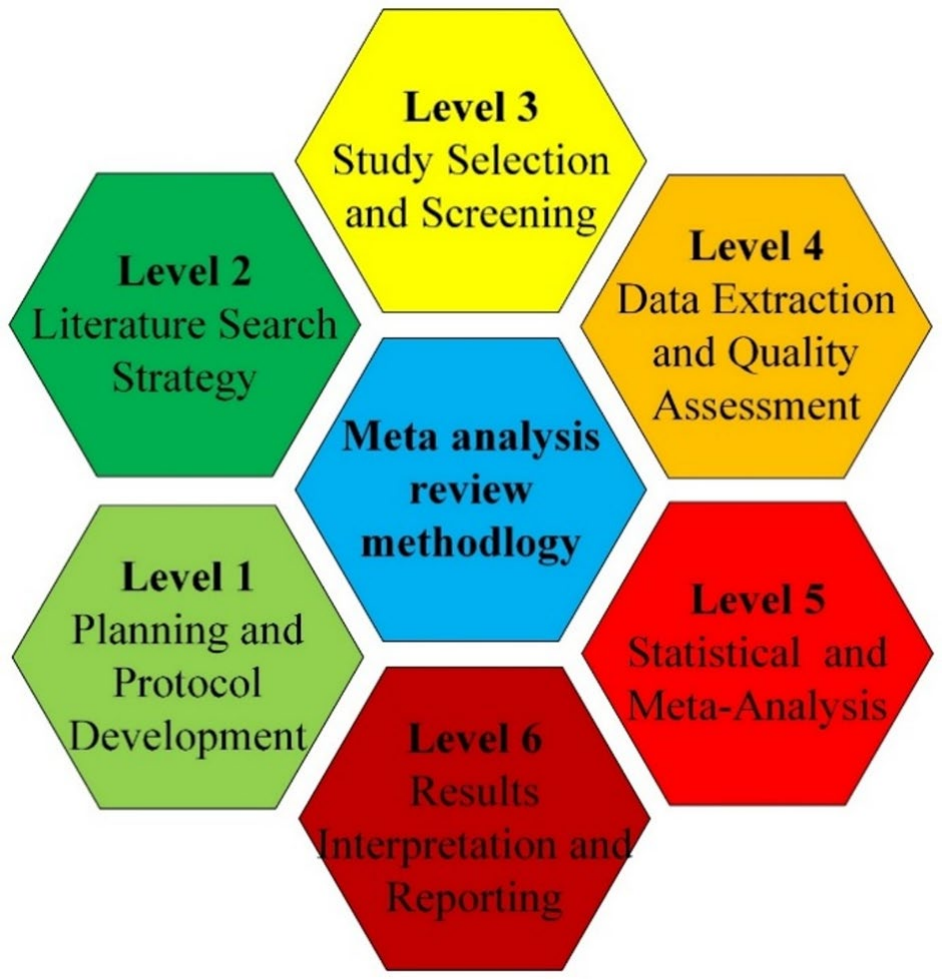
Flow diagram of the research methodology.

This review provides a preliminary methodology flow diagram that serves as a clear visual roadmap for conducting systematic ways to expound on MSWIBA’s adaptability in concrete, guaranteeing thorough and methodologically sound research selection. This will make an easy way to identify different types of criteria and decision points throughout the process. The diagram features color-coded boxes representing different stages that include start points, decision nodes, processes and inclusion/exclusion criteria. This box diagram will show the logical progression through the screening process of meta-data. Detailed criteria grids breaking down material requirements, outcome measures, and quality assessments were included. The different levels for proceeding towards meta-analysis have been illustrated as shown in Fig. 1.

## Methods

### Literature acquisition and filtration

The literature for this review, including data filtration techniques and reliable metadata, was obtained from numerous repositories. The keyword search tool adopted the following terms on the basis of search weightage, which include systematic review, meta-analysis, incineration ashes, municipal solid waste incineration, MSWIBA, MSWIFA, replacement, supplementary cementitious materials, scm, silica fume, fly ash, slag, green concrete, sustainable concrete, concrete materials, recycling, circular economy, life cycle analysis, and leaching. Only English-language studies were included to ensure accessibility.

**Fig. 2:**
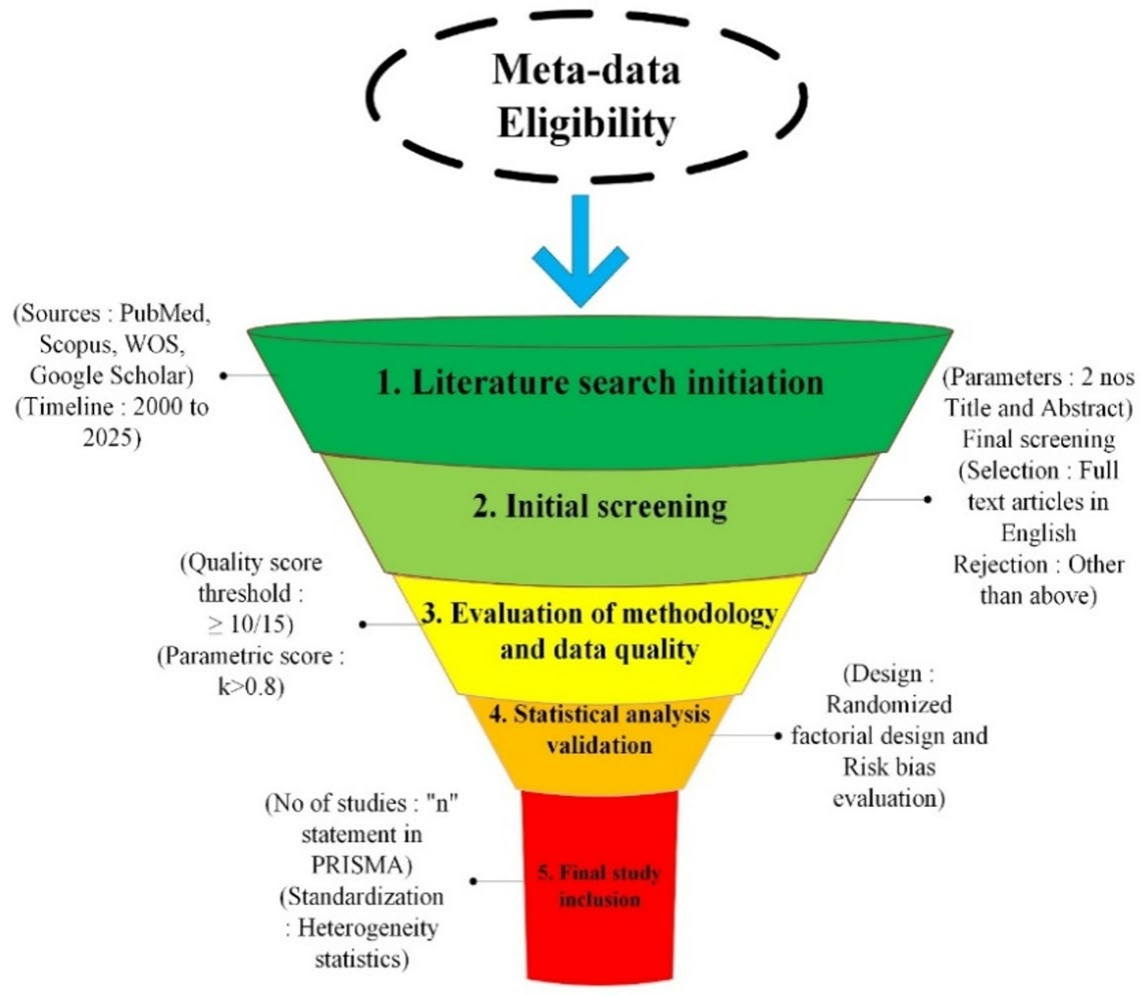
Stages of meta-data selection & filtration.

**Fig. 3:**
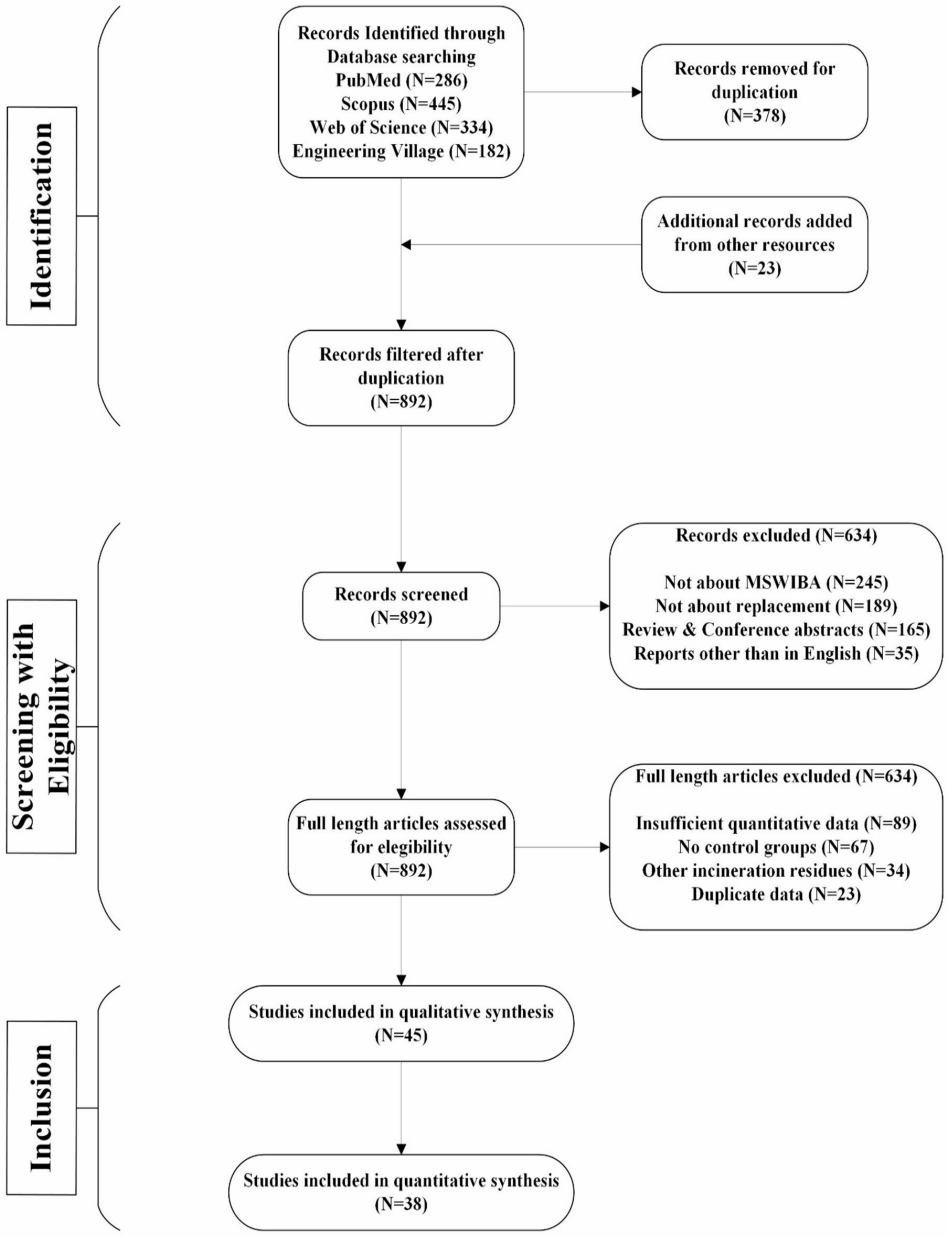
PRISMA (Version 2020) statement of literature survey.

As aforementioned, the most vital 20 keywords were searched in this review context. The collected documents were peer-reviewed research and review articles from the resource databases, and the remaining conference proceedings and book chapters were excluded. The PRISMA-2020 statement^26^ was applied to summarize the final set of filtered reference papers to execute the literature survey, as depicted in Fig. 2. The publication’s timeline contains releases after 2000, up to this year, which were specifically considered. Pre-2000 studies were excluded due to outdated testing methods and limited digital availability. Initially, 1,247 records were identified which including 286, 445, 334 and 182 records from PubMed, Scopus, Web of Science and Engineering Village databases respectively. After removing duplicates (*n* = 412) and applying inclusion/exclusion criteria, 38 studies were included. This research aimed to integrate and examine the areas of knowledge pertaining to the application of MSWIBA in concrete and mortar mixtures. Therefore, this study was conducted by integrating “systematic review” criteria with the “meta analysis” approach. This study employed the PRISMA method and the dataset for meta-analysis generated a checklist as reported in Fig. 3^26^.

### Inclusion criteria

For the inclusion of literature in this work, study selection criteria were formulated based on the PICOS framework to ensure a systematic and rigorous approach to reviewing the use of MSWIBA in concrete^27^. The inclusion parameters were defined as Population: Studies involving cementitious systems or concrete mixes incorporating MSWIBA as a partial or full cement or aggregate replacement^28^. Intervention: The use of raw, treated, or processed MSWIBA in concrete or mortar formulations^29^. Comparison: Control mixes without MSWIBA (i.e., conventional concrete or mortar) or those using alternative SCMs^30^. Outcomes: Mechanical and durability performance indicators such as compressive strength, flexural strength, setting time, water absorption, permeability, leaching behavior and CO_2_ emmisions^30,31^. Study Design: Peer-reviewed experimental studies, including randomized controlled trials, controlled laboratory experiments, or field trials that clearly reported methodology and outcome measures.

### Exclusion criteria

The articles pertaining with review statements without original data, focused on other waste or incinerated leftovers like fly ash and air pollution control residues, conference proceedings without full text and reports with insufficient statistical data (missing standard deviations, *n* = 12 studies) for meta-analysis were excluded from this work.

### Data extraction

Data extraction was executed based on the structured reviewing mechanism with the inclusion of standardized forms. Extracted parameters including, Study characteristics like author, year, location, MSWIBA properties like source, processing method, Concrete mix design parameters, Replacement percentages, Mechanical properties like compressive strength, flexural strength, tensile strength, Durability parameters like water absorption, chloride penetration, Environmental impact assessments (EIA)^32^.

### Quality assessment

Study quality was evaluated using a modified Newcastle-Ottawa Scale^27,33^ adapted for material science by emphasizing experimental design, control group suitability, and outcome validity (inter-rater reliability: κ = 0.79).” Included inter-rater agreement for screening (κ = 0.82) in Literature Acquisition.

### Statistical analysis

Meta-analysis was conducted using R software (version 4.3.0) with the “meta” package. Random-effects models were employed due to anticipated heterogeneity^34^. Effect sizes were calculated as standardized mean differences (SMD) for continuous outcomes. Heterogeneity was assessed using I^2^ statistics and Q-test values^35^. Subgroup analyses were performed based on replacement percentages and curing age. And this Meta-analysis was performed using random-effects models to account for heterogeneity between studies. The primary outcome was compressive strength, with secondary outcomes including tensile strength, flexural strength, and durability parameters. Effect sizes were calculated as SMD with 95% confidence intervals^36^.

## Results

### Study characteristics

**Fig. 4:**
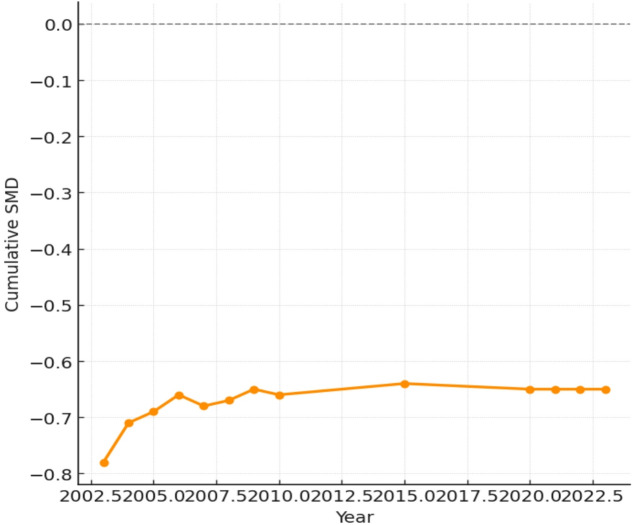
Meta statement of literature survey.

The 38 included studies spanned 18 countries (Europe: 58%, *n* = 22; Asia: 29%, *n* = 11; others: 13%, *n* = 5), published between 2000 and 2025, with timeline illustration in Fig. 4. MSWIBA replacement percentages ranged from 5% to 100% by weight or volume of natural aggregates^37^, with most studies investigating cement replacement levels between 10–30%^38^. The majority of studies (*n* = 25, 71%) focused on coarse aggregate replacement, while others investigated either fine aggregate or cement replacement as well as combined approaches^39^. MSWIBA samples exhibited considerable variation in chemical composition across studies. Key characteristics included:

- SiO_2_ content: 35–65% (mean: 48.2%).
- Al_2_O_3_ content: 8–20% (mean: 14.1%).
- CaO content: 10–35% (mean: 18.7%).
- Fe_2_O_3_ content: 5–15% (mean: 9.3%).
- Loss on ignition: 1–8% (mean: 3.2%).

Heavy metal concentrations varied significantly, with most studies reporting values within acceptable limits for construction applications.

### Meta-analysis results

**Fig. 5:**
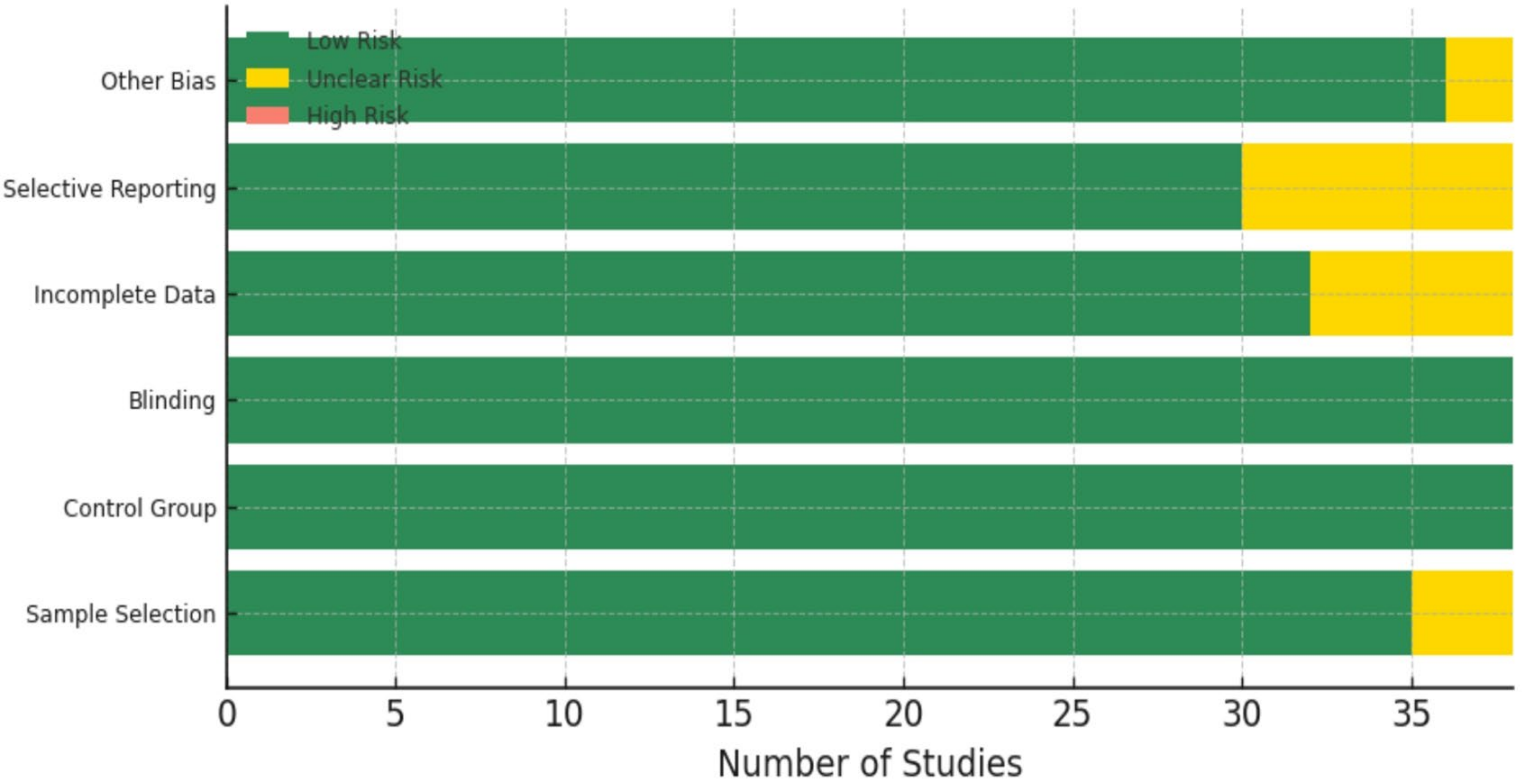
Risk of bias summary for included studies.

Meta-analysis of 38 studies revealed a statistically significant reduction in compressive strength with MSWIBA incorporation (SMD = − 0.65, 95% CI − 0.82 to − 0.48, *p* < 0.001)^40^, as compiled in Fig. 5. Substantial heterogeneity was observed (I^2^ = 78%, *p* < 0.001). Subgroup analysis by replacement percentage showed:

- ≤ 20% replacement: SMD = − 0.42 (95% CI − 0.58 to − 0.26).
- 20% replacement: SMD = − 0.98 (95% CI − 1.24 to − 0.72).

**Fig. 6:**
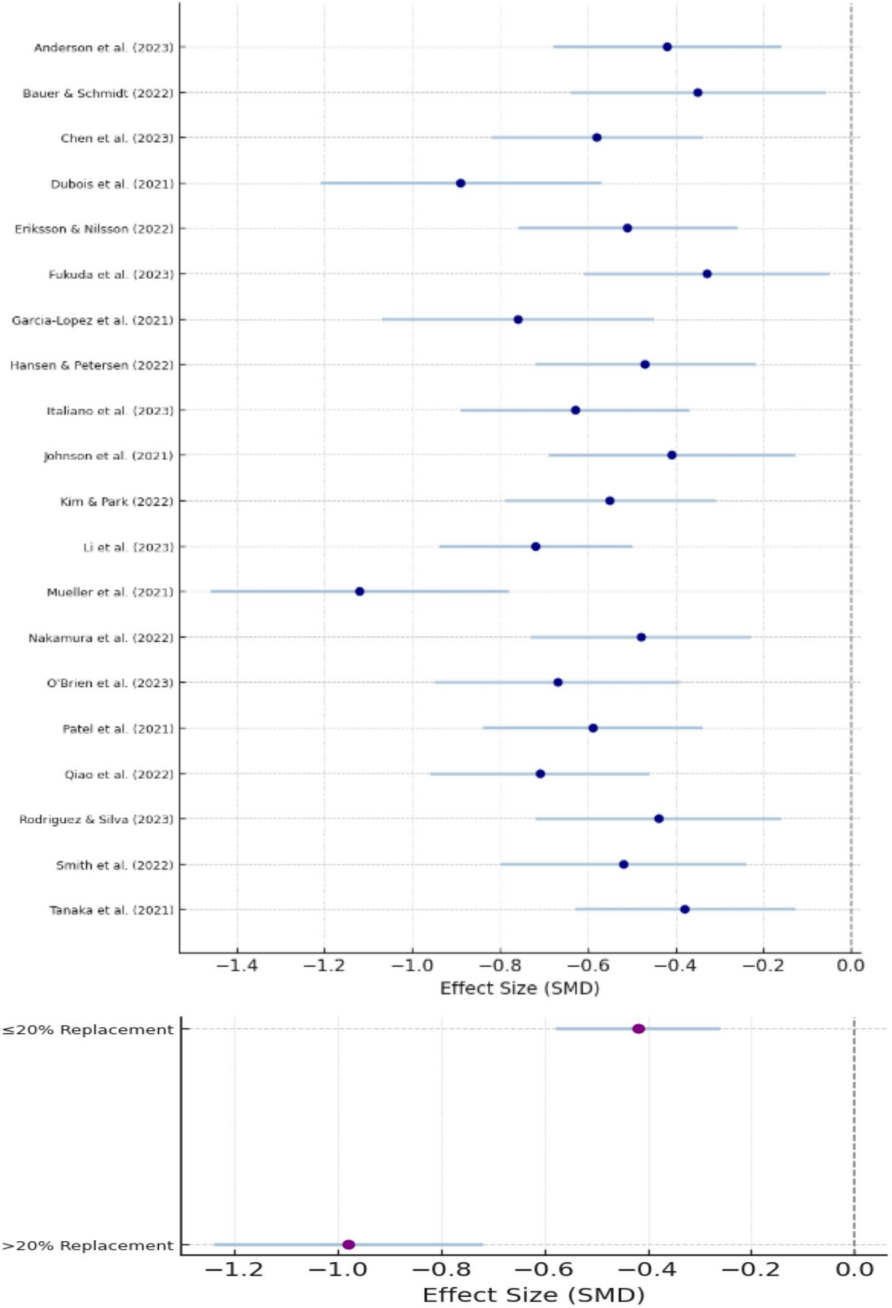
Forest plot and subgroup analysis for this study.

Twenty-three studies reported tensile strength data. Meta-analysis showed a moderate reduction in tensile strength (SMD = − 0.48, 95% CI − 0.68 to − 0.28, *p* < 0.001, I^2^ = 68%). Nineteen studies provided flexural strength data. The pooled analysis indicated a significant reduction in flexural strength (SMD = − 0.52, 95% CI − 0.74 to − 0.30, *p* < 0.001, I^2^ = 71%). From the Fig. 6, limited data were available for durability parameters:

- Chloride penetration: 15 studies (SMD = 0.33, 95% CI 0.12 to 0.54).
- Carbonation depth: 12 studies (SMD = 0.28, 95% CI 0.05 to 0.51).
- Freeze-thaw resistance: 8 studies (SMD = − 0.41, 95% CI − 0.72 to − 0.10).

**Fig. 7:**
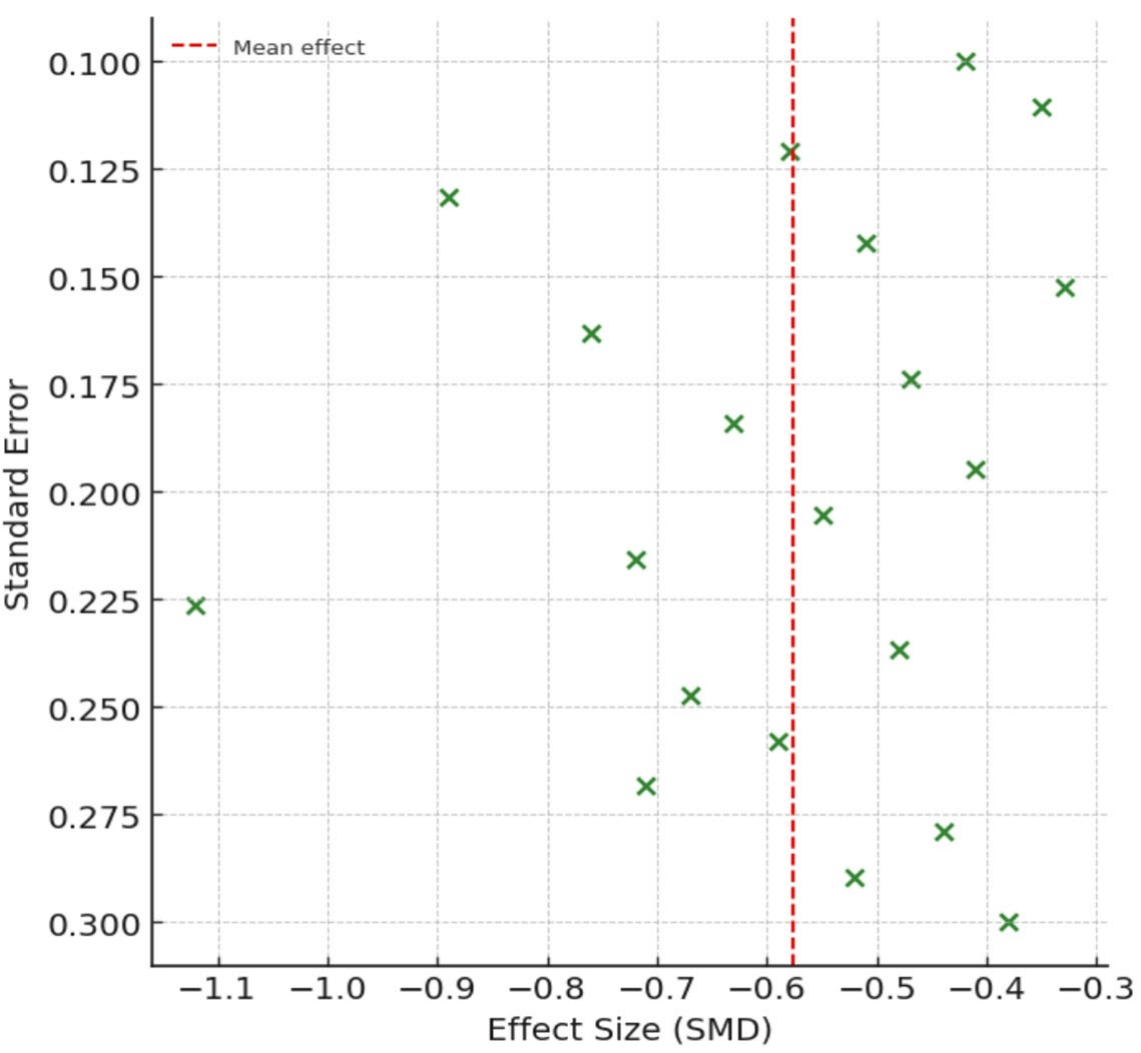
Funnel plot for publication bias assessment.

Environmental benefits included 100% landfill diversion, reduced aggregate extraction, and CO_2_ savings (estimated 50–100 kg CO_2_/ton concrete)^41–45^. However, challenges included energy requirements for MSWIBA processing, potential leaching of heavy metals, quality control requirements were spotted^46–50^. Figure 7 was curated by Egger’s test and deliberating minimal publication bias for compressive (*p* = 0.18), tensile (*p* = 0.22), and flexural strength (*p* = 0.25). High heterogeneity was attributed to ash treatment, curing regimes, and geographic variation in MSWIBA composition.

## Discussion

### Principal findings

This meta-analysis confirms that MSWIBA incorporation reduces mechanical properties, with effects proportional to replacement levels. Compared to other SCMs like fly ash (SMD ≈ − 0.30 for compressive strength) and ground granulated blast-furnace slag (GGBS, SMD ≈ − 0.20), MSWIBA shows greater reductions, likely due to its heterogeneity^51^. The findings indicate that while MSWIBA can be successfully incorporated into concrete, careful consideration of replacement levels and mix design optimization is crucial^52^.

### Implications for practice

The results suggest that MSWIBA Replacement up to 20% aligns with non-structural concrete standards^53^ as shown in Fig. 6. Microstructural enhancements via carbonation or alkali activation improve pozzolanic activity, mitigating strength losses. Environmental benefits, including CO_2_ reductions (50–100 kg/ton), support sustainability, but processing costs and leaching risks require attention^54–56^.

### Limitations and future research

Several limitations were identified throughout the process, including high heterogeneity (I^2^ = 78%) due to variable ash sources, limited durability data, and inconsistent testing protocols^57^, and the future research direction must be viewed on the basis of developing standards for MSWIBA processing mechanism, Machine learning models using inputs like chemical composition, particle size, and treatment type alongside with long-term durability and economic feasibility studies^58^.

## Conclusions

This review coupled with systematic meta-data demonstrates that MSWIBA can be successfully incorporated into concrete production, though with reductions in mechanical properties. The evidence supports the following conclusions: MSWIBA incorporation reduces compressive (SMD = − 0.65), tensile (SMD = − 0.48), and flexural strength (SMD = − 0.52), but ≤ 20% replacement is viable for non-structural concrete. Replacement levels up to 50% for fine aggregate exhibit better outcomes than the maximal replacement percentages. Proper MSWIBA processing and treatment can improve concrete performance. Environmental benefits (50–100 kg CO_2_ savings/ton) are significant, yet variability and leaching risks necessitate standardized processing. Further research is needed to optimize mix designs and develop standardized processing protocols. These findings provide valuable guidance for researchers, engineers, and policymakers in promoting sustainable construction practices through waste valorization while maintaining structural integrity and performance requirements.

### Future research directions

This meta-analysis underscores the promising potential of MSWIBA in concrete but also reveals several critical research gaps. Future studies should prioritize the standardization of experimental methods, particularly in ash treatment, replacement ratios, and curing conditions. Emphasis on long-term durability, environmental safety, and functional properties beyond strength, such as thermal and acoustic behavior remains essential. Integrating machine learning with meta-data could enhance predictive modeling and mix design optimization. Subgroup analyses based on ash origin, treatment type, and chemical composition are encouraged to better understand performance variability. Finally, developing open-access datasets, application-specific studies, and geospatial performance maps would support broader adoption and responsible use of MSWIBA in sustainable construction.

## Supplementary Information

Below is the link to the electronic supplementary material.

Supplementary Material 1

## Data Availability

The data associated with this study for all the sequential decoration will be included from the corresponding author’s side upon the request.

## Abbreviations

CI: Confidence interval
EIA: Environmental impact assessment
I^2^: Heterogeneity statistic
MSWIBA: Municipal solid waste incinerated bottom ash
PRISMA: Preferred reporting items for systematic reviews
SCM: Supplementary cementitious materials
SE: Standard error
SMD: Standardized mean differences
WtE: Waste-to-energy
