# Supplementary material for: Municipal solid waste incineration bottom ash in concrete : A systematic review and meta-analysis
Source: Sci Rep. 2025 Nov 14;15:39931. doi: 10.1038/s41598-025-23774-6 (PMC12618590; doi:10.1038/s41598-025-23774-6)
Supplement: Supplementary file 1 — Supplementary Material 1 [file 41598_2025_23774_MOESM1_ESM.docx]

**Supplementary data of**

A manuscript entitled **“Adaptation of Municipal Solid Waste Incineration Bottom Ash in Concrete : A Systematic Review and Meta Analysis”** (Manuscript ID: e0fb953c-ea6b-45c7-bbe4-e3569367f760 v2.0) has been submitted to the journal *Scientific Reports* by Mr. Sivayogaraj A & Dr. Elavenil S from the School of civil engineering, Vellore institute of technology university chennai campus – 600127, Tamilnadu, India.

**Supplementary Table S1: Search Strategy Details**

| Database | Search String | Filters | Results |
| --- | --- | --- | --- |
| PubMed | ((("Municipal solid waste"[Title/Abstract] OR "MSW"[Title/Abstract]) AND ("incineration"[Title/Abstract] OR "incinerated"[Title/Abstract]) AND ("bottom ash"[Title/Abstract] OR "IBA"[Title/Abstract])) OR "MSWIBA"[Title/Abstract]) AND ("concrete"[Title/Abstract] OR "mortar"[Title/Abstract] OR "cement"[Title/Abstract]) | English, 2000-2025, Journal Articles | 286 |
| Scopus | TITLE-ABS-KEY(("municipal solid waste" OR "MSW") AND ("incineration" OR "incinerated") AND ("bottom ash" OR "IBA") OR "MSWIBA") AND TITLE-ABS-KEY("concrete" OR "mortar" OR "cement") | English, 2000-2025, Articles | 445 |
| Web of Science | TS=(("municipal solid waste" OR "MSW") AND ("incineration" OR "incinerated") AND ("bottom ash" OR "IBA") OR "MSWIBA") AND TS=("concrete" OR "mortar" OR "cement") | English, 2000-2025, Articles | 334 |
| Engineering Village | ((("municipal solid waste" OR "MSW") AND ("incineration" OR "incinerated") AND ("bottom ash" OR "IBA")) OR "MSWIBA") WN ALL AND ("concrete" OR "mortar" OR "cement") WN ALL | English, 2000-2025, Journal Articles | 182 |
